# Comprehensive strategy for capturing and integrating community input into community research training curricula

**DOI:** 10.1017/cts.2018.11

**Published:** 2018-07-06

**Authors:** Jennifer Cunningham-Erves, Yvonne Joosten, Marino A. Bruce, Jared Elzey, Patrick Luther, Lexie Lipham, Yolanda Vaughn, Tonya Micah, Consuelo H. Wilkins, Stephania T. Miller

**Affiliations:** 1 Meharry Medical College, Nashville, TN, USA; 2 Vanderbilt University Medical Center, Nashville, TN, USA; 3 Vanderbilt University, Nashville, TN, USA; 4 Nashville Cares, Nashville, TN, USA; 5 Neighborhoods Resource Center; 6 Meharry-Vanderbilt Alliance, Nashville, TN, USA

**Keywords:** Community research capacity building, community engagement, curriculum development, research training, community populations

## Abstract

**Introduction:**

Community stakeholders often participate in community research training curricula development. There is limited information describing how their input informs curricula. This paper describes input solicitation methods, input received, and examples of its integration.

**Methods:**

From June 2014 to June 2016, community members (CMs) and community-based organizations (CBOs) guided curricula development tailored for CMs and CBOs, respectively. Engagement methods included a strategic planning retreat, surveys, a listening session, workgroup meetings, and community engagement studios. Descriptive statistics were used to summarize survey input. For other methods, input was extracted and compiled from facilitator notes.

**Results:**

CMs (n=37) and CBOs (n=83) providing input included patients and caregivers and advocacy, community service, and faith-based organizations, respectively. The major feedback categories were training topic priorities, format (e.g., face-to-face vs. online), logistics (e.g., training frequency), and compensation (e.g., appropriateness). Input directly guided design of CBO and CM curricula (e.g., additional time devoted to specific topics based on feedback) or helped to finalize logistics.

**Conclusions:**

Multiple quantitative and qualitative methods can be used to elicit input from community stakeholders to inform the development of community research training curricula. This input is essential for the development of training curricula that are culturally relevant and acceptable.

## Introduction

Community-engaged research (CEnR) is an overarching framework that leverages academic and community strengths to improve community health and well-being [1–4]. A fundamental principle of CEnR is that community is engaged in all phases of the research process [5, 6]. Other principles include equitably partnering with community to address their problems and concerns, acknowledging community diversity, developing community capacity for meaningful engagement, and committing to long-term engagement [6].

The Meharry-Vanderbilt Community-Engaged Research Core (CERC), supported by the Meharry Clinical and Translational Research Center and the Vanderbilt Institute for Clinical and Translational Research, leverages academic and community strengths to improve community health. This core is guided by the CERC Community Advisory Council, which was formed in 2007 to promote the utility and value of CEnR to academic and community stakeholders. The Mini-grant has been a key program during this period as a number of community-based organizations (CBOs) have received support to collaborate with academic researchers to collect pilot data on community-identified health issues. Early program evaluation highlighted the need for CBOs to develop and expand their capacity to engage in research. As a result, CERC developed stand-alone research training sessions (e.g., Research 101) and full-day research training forums (e.g., research agenda development) with input from participating CBOs and its Community Advisory Council. This work, over several years, laid the foundation for the development of two related, but distinct, research training curricula: one for CBOs, and another for community members.

The Community Research Capacity-Building Team was formed to develop and implement research training modules and is comprised of individual community members, CBO representatives, and academic faculty and staff from Meharry Medical College and Vanderbilt University Medical Center. It has been established that the inclusion of community members and CBOs in curriculum development is essential for the development of materials that are relevant and acceptable for community stakeholders [7]. But, the scholarly literature is less clear about the *type* of input gathered and the process for integrating community stakeholder ideas and suggestions into the final training programs. These gaps are particularly salient when developing and/or tailoring training programs that are responsive to community needs. Soliciting and describing the *type* of input may illuminate unique aspects of training that are important to community stakeholders. A description of *how* the input was incorporated may be informative to others interested in developing and/or tailoring their community research training programs. The objective of this paper is to describe the CERC Community Research Capacity-Building Team’s curricula development process, including the type of input received from community stakeholders and examples of how it was integrated into separate research training curricula for CBOs and community members.

## Methods

**Fig. 1:**
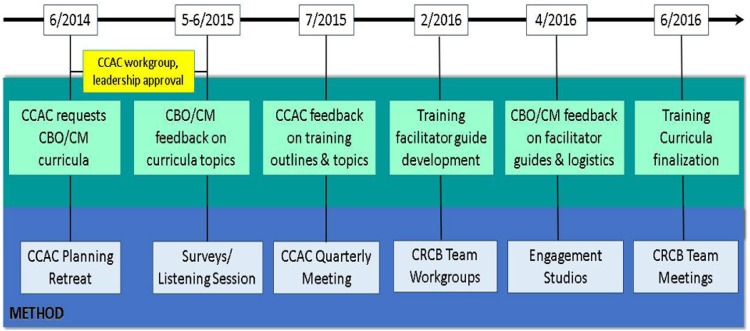
Community-engaged community research curricula development process. For the noted time periods, various methods were used to solicit and iteratively integrate community stakeholder feedback into community research training curricula. CCAC, CERC Community Advisory Council; CERC, Community-Engaged Research Core; CBO, community-based organization; CM, community members; CRCB, Community Research Capacity-Building.

An iterative, community-engaged curricula development process was implemented over 2 years. Though we were aware, via our literature searches, of existing community research curricula, we wanted to ensure that each curriculum reflected the specific needs of our community stakeholders. The CBO curriculum goal was to increase organizations’ capacity to conduct research independently and in collaboration with academic researchers. The community member curriculum goal was to increase individuals’ capacity to serve as research team members and in advisory roles, such as advisory board members. Each phase of the development process and the feedback received at each phase are described below and outlined in Fig. 1.

### Phase I: Community Advisory Council Strategic Planning Retreat—June 2014; Post Retreat Workgroup—September 2014 to February 2015

After several years of promoting CEnR, the CERC Community Advisory Council held a 1-day strategic planning retreat to assess CERC’s progress and effectiveness and to set the agenda for the next 2 years. With the help of an independent community facilitator, the advisory council members reviewed all CERC activities and developed a list of recommendations to enhance existing activities and to start new ones. These recommendations were intended to increase the presence and strengthen the role of patients and other community stakeholders in all research phases. The retreat facilitator captured all feedback in a final report. Following the retreat, the CERC Community Advisory Council appointed a workgroup to refine and organize the feedback into priority outcomes, metrics, and recommendations for future CERC activities.

### Phase II: Community-Based Organization Surveys—May and June, 2015

CBOs were surveyed to capture their research training interests and needs. A Research Electronic Data Capture (REDCap) [8] survey was sent (May 2015) to CBO representatives that had participated in at least one CERC-sponsored community research capacity-building activity, such as a community research forum. The forums, for which community stakeholders played an integral role in planning, implementing, and disseminating results [9], are workshops designed to help CBOs define, develop, and implement their own research agendas. The survey asked CBO representatives to indicate preferences for various training topics (e.g., Research 101—see Results section for full response option list). Feedback gleaned from the aforementioned CERC Community Advisory Council retreat, previous training needs assessments [9], and consultation requests informed the training topics. They were also asked to identify preferences for training format (e.g., face-to-face vs. online) and logistics (e.g., time, location, frequency).

For input from a broader CBO audience about curricula research topics, another REDCap survey was distributed (June 2015) through CERC’s Community Research Partners electronic newsletter. The newsletter reaches over 300 CBOs, including those that had attended and/or presented at CERC’s monthly community research partner meetings or attended community research forums. Five additional response options were added to the initial survey (e.g., collaborating with an academic partner). Descriptive statistics, embedded within the REDCap interface, were calculated for all CBO survey responses.

### Phase III: Community Expert Listening Session—June 2015

Community members with previous research advisory experience were invited to participate in a listening session to reflect on their experiences, learn about other research advisory role opportunities, and provide recommendations to improve their research experience. They had previously served as community and patient stakeholders or “community experts” in CERC community engagement (CE) studios. A CE studio is a consultative model that provides project-specific input from community experts to researchers [10]. During the listening session, CERC facilitators asked a group of community experts to identify what types of research support or training would be most helpful to them. Based on their verbal responses, CERC facilitators prepared a written summary of the listening session.

### Phase IV: Community Advisory Council Quarterly Meeting—July 2015

A quarterly Community Advisory Council meeting was used to elicit council member input on initial curricula drafts. Based on survey feedback and community listening sessions above, from June to September 2015, the Community Research Capacity-Building team drafted the outlines for CBO and community member curricula. The outlines included proposed objectives, topics, deliverables, and facilitators. During the quarterly meeting, input was captured in meeting notes and written notes.

### Phase V: Community Research Capacity-Building Team Workgroups (Facilitator Guide Development)—September 2015 to April 2016

From September 2015 to February 2016, the Community Research Capacity-Building team’s CBO representatives and academic faculty and staff held monthly face-to-face workgroup meetings to expand the initial curricula outlines into trainer facilitation guides. This included identifying and comparing existing training guides that could be adapted for our purposes. We decided on one developed by the Tufts Clinical and Translational Science Institute as part of their community research capacity-building efforts [11], which included the following sections: (1) Session Summary; (2) Learning objectives; (3) Preparation work; (4) Proposed activities; and (5) Additional resources.

### Phase VI: Community Engagement Studios—April 2016

To gather detailed feedback on the draft trainer facilitator guides, one CE studio was conducted with previous CE studio experts, community members, and patients. A second CE studio enlisted CBO representatives. Prior to the CE studios, both groups received the draft trainer facilitator guides to review. During the studio, following additional review time, the facilitator solicited general comments about the guides and specific thoughts about various sections. Both groups were also asked about logistics, timing, and incentives for participation. The CE studio experts were asked to share their preference on either having an initial “Research 101” training session with community members only or one in combination with CBOs. This, along with determining an appropriate compensation amount, had been a topic for which the Community Research Capacity-Building Team could not reach a consensus. In keeping with standard CE studio procedures [10], the facilitator recorded CE studio expert and CBO verbal responses and key points on flip charts that were visible to all. Specific preferences (e.g., time of day for training) were decided by majority vote. Because the CE studio is a consultative model and not research (participants are not research subjects), their responses were summarized in a written facilitator report rather than undergoing qualitative analysis.

### Phase VII: Community Research Capacity-Building Team Meetings (Curricula Finalization)—April to June 2016

The curricula facilitator guides, including training session topics, learning objectives, and other aspects of the training (e.g., training format, logistics) were finalized through the monthly Community Research Capacity-Building Team meetings and electronic communications as needed.

## Results

A multistep, community-engaged process was used to develop the research training curricula for CBOs and community members. The type of feedback and examples of how it was incorporated into the curricula are summarized below and in Table 1.

**Table 1:** Community stakeholder curricula feedback and integration into research curricula

Feedback received	How feedback was incorporated
Community Advisory Council Strategic Planning Retreat	
∙ Build capacity of community leaders and organizations to engage in research ∙ Develop curricula tracks for CMs and CBOs	∙ Initiated efforts to develop separate research curricula for CMs and CBOs. See additional subsequent efforts below
Community-based organization surveys (training topics)	
*In order of interest*: ∙ Applying for federal research grants; Research 101; database management; research methods; developing a research budget; manage research grant; conducting literature review	∙ All topics* included subsequent curriculum feedback requests ∙ All topics* retained in the final CBO curriculum
Community-based organization surveys (training format)	
*In order of preference*: ∙ Group, face-to-face ∙ Independent, online ∙ Live webinar	∙ Group, face-to-face format highlighted in subsequent curriculum feedback requests ∙ Group, face-to-face format used for final CM and CBO curricula training sessions
Community expert listening session (training topics)	
*Provide training on following topics*: ∙ Research ethics ∙ Funding ∙ Dissemination	∙ All topics* highlighted in subsequent curriculum feedback requests ∙ All topics* retained in the final CM curriculum
Community Advisory Council quarterly meeting (training outline guidance)	
∙ Integrate community perspective in training ∙ Include a community representative, with research experience, as a training co-facilitator for CM curriculum	∙ Community co-facilitators, with research experience, identified ∙ Community co-facilitators presented during CBO and CM curricula implementation
Community member CE studio (content/guidance for facilitator guides)	
∙ Provide a comprehensive training packet ∙ Include optional prework (e.g., readings, videos) ∙ Combine CM and CBO trainees for the first session, “Research 101” ∙ Allocate less time to developing research questions	∙ Training packet handed out at first session and supplemented during subsequent ones ∙ Optional prework included per session ∙ Research 101 session included CBO representatives and CMs ∙ Research question development highlighted in one (“Research 101”) of 3 CM training sessions
Community member CE studio (training frequency and duration)	
∙ Preference for weekly sessions ∙ Ideal duration—average of 2 h	∙ Weekly, 2.5-h research training sessions implemented as part of final CM curriculum
CBO CE studio (content/general recommendations based on facilitator training guides	
∙ CBO trainees should get feedback from their own stakeholders about research priorities before starting the training ∙ Allocate more time to the development of research questions	∙ CBO trainees encouraged pretraining stakeholder t to meet CBO research needs ∙ Research question development exercises were highlighted or integrated into 3 (“Research 101”) of 6 CBO training sessions
Community-based organization CE studio (training frequency/day)	
∙ Preference for biweekly sessions ∙ Preference for Tuesdays/Wednesdays	∙ Biweekly CBO research training sessions implemented on Wednesdays
Community-based organization and community member CE studio (compensation)	
∙ Only some CMs and all CBOs felt compensation was appropriate	∙ Compensation ($50) was provided to CMs and CBO representatives

CM=community member; CBO=community-based organization; CE=community engagement.

* Integrated as focus of overall training session or as separate training session component.

### Community Advisory Council Strategic Planning Retreat

A total of 11 CERC Community Advisory Council members participated in the retreat. They represented community coalitions, advocacy organizations, public health, faith-based organizations, community health centers, and social service providers. They prioritized the need to build the research capacity of community leaders and organizations. One member stated, “Build the capacity of community organizations and community leaders to be collaborating partners in community-engaged translational research.” The post retreat workgroup integrated the retreat feedback into specific recommendations for curriculum objectives: (1) increase community-driven research; (2) increase the participation of underserved populations in translational research; (3) empower community stakeholders to reduce system barriers to community participation in research; and (4) increase the number of community members on research teams and academic publications. Advisory council members also recommended that training resources be targeted to both CBOs and individual patients or community members. This guided the development of 2 research curricula—1 for community members and 1 for CBOs.

### Community-Based Organization Surveys

**Fig. 2:**
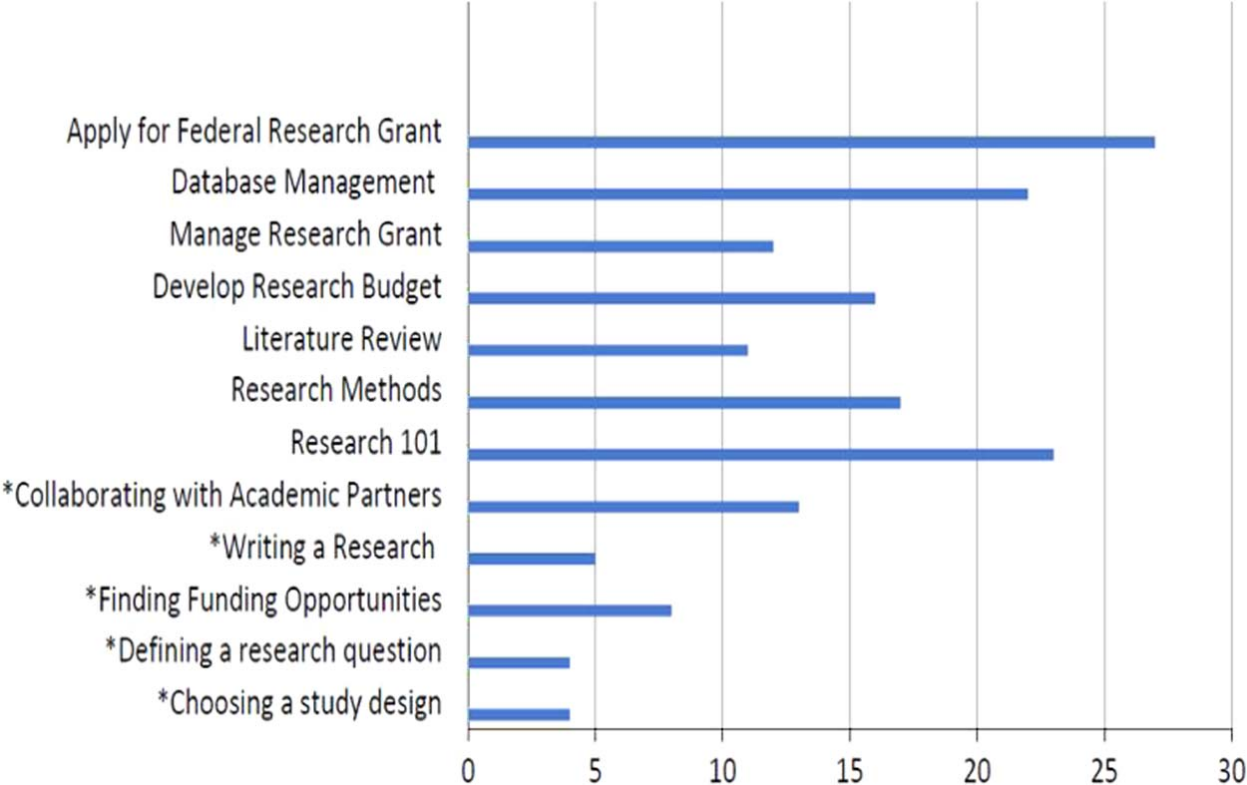
Training priorities of community-based organizations (CBOs). Using 2 separate online surveys, CBO representatives (n=51; 32 for survey 1, 19 for survey 2) identified research training priorities for their organizations. *These priorities were identified on survey 2 only.

In total, 51 CBO representatives from advocacy, health social services, faith-based, neighborhood, and government agencies provided their research training preferences via the 2 online REDCap surveys described above. “Applying for federal research grants” ranked as the most desired topic (27, 19%) followed by Research 101 (23, 16%). See Fig. 2 for all topics. Among those who completed the first survey (n=32), 27 responded to the question about training format preferences. There was slightly greater preference for a group, face-to-face format (20, 74.1%), compared with an online training module that could be accessed anytime (19, 70.4%).

For the 5 additional research topics that were included on the second CBO survey only (n=19) (data not shown), the training topic of greatest interest was “Collaborating with Academic Partners” (13, 68.4%) followed by “Writing a research proposal” (5, 26.3%). See Fig. 2 for all topics.

All topics from both CBO surveys were either included as a session component in the final CBO curriculum, or given a dedicated session. For example, though there was not a session that focused on “Choosing a Study Design,” this topic was covered in “Research Methods.” Based on CBO preferences and additional resources needed for online implementation, training was conducted face-to-face.

### Community Expert Listening Session

In total, 30 community members attended the community listening session. All had participated in at least 1 CE studio and represented patients with a variety of health conditions, caregivers, and different geographic and racial communities. Most expressed interest in serving in different research roles including research team member, grant reviewer, or research advisory council member. They felt that receiving training in the research process would make them more effective in these roles. There was consensus on topics requested: research ethics, funding, and dissemination of research results. These were incorporated into training sessions specific for community members.

### Community Advisory Council Quarterly Meeting

The CERC Community Advisory Council’s (n=11) primary recommendation on the initial training outline was to consider the community perspective on highlighted research topics and how it complements and differs from the academic perspective. To this end, they recommended using specific community facilitators to deliver the training. These recommendations were consistent with initial decisions by CERC’s Community Research Capacity-Building Team to include community co-facilitators. Accordingly, members of our Community Research Capacity-Building Team, co-authors PL and YV, were included among community facilitators that were identified based on their insight and expertise (e.g., finding CBO funding opportunities).

### Community Engagement Studios

The community member CE studio focused on the community member curriculum facilitator guide, training format, and logistics. Seven community members served on the expert panel and represented a range of experience in research advisory roles, including no experience, 1 or more CE studios, research advisory council member, and member of a research team. They were enthusiastic about the opportunity to learn more about research methods and potential research advisory roles. They liked the ideas of including interactive exercises as part of the training sessions and participating in the first “Research 101” training session with CBOs. However, there were some concerns about the intensity of some of the sessions, the time commitment, and use of technical research terms. Much of the related feedback and recommendations addressed these concerns. For example, a content-related suggestion was to reduce the time devoted to developing research questions since they felt that community members, unlike CBOs, were less likely to be involved in developing research questions. They also suggested ways to keep up with all the research training materials (e.g., include a comprehensive training packet). Though there were divergent preferences on training session times (weekends vs. weekdays), they all agreed that weekly sessions, versus biweekly, would be best for knowledge retention. There was general agreement that around 2 hours/training session would be ideal, but that some topics might require more time. There was no consensus on monetary compensation for trainees, with some indicating they would participate without compensation and while others felt it was necessary. We integrated all these suggestions into the community member curriculum and made the decision to offer each trainee $50/completed training session.

In total, 10 CBO representatives participated in the CE studio, advising on the CBO curriculum facilitator guide and other training implementation. Faith-based, grassroots community advocacy, mental health, funding, and neighborhood organizations were represented. Panel members’ research-related roles within their organization ranged from developing surveys, collecting and managing data, developing partnerships with academic researchers, managing research partnerships, and grant writing, to no previous research-related activities. The experts thought the training would expand their skills and increase research activity at their organization. Several felt that it would be helpful to convince CBO leadership that this training was valuable to the organization. One of the related recommendations was for trainees to get organizational stakeholder input about the CBO’s research goals before the training. Unlike community members, they suggested that more time be devoted to developing research questions. There was a preference for biweekly sessions on Tuesdays or Wednesdays. There was general agreement that compensation was appropriate, but not on the amount. CBO representatives could not decide if it would be appropriate for a CBO trainee to receive compensation if they were attending on behalf of their CBOs through which they were already being paid for their time. We incorporated all recommendations, including setting the compensation amount at $50/completed session with CBO trainees having the option to either keep the compensation for themselves or donate it back to their CBOs.

### Completed Curricula

This community-engaged curriculum development process produced 2 curricula tracks: (1) for individual community members who are interested in serving in research advisory or consulting roles and (2) for representatives of CBOs interested in partnering with researchers or conducting their own research (Table 2). Each track included facilitator guides suggesting session preparation work, session activities, and additional resources (not shown) for which the facilitators would have autonomy in modifying as needed or desired.

**Table 2:** Finalized community research curricula training topics and learning objectives

Community-based organization training session learning objectives
Session 1: Introduction to research
Define and describe research (e.g., question, hypothesis, aims, methods, etc.).
Describe research process steps.
Identify translational research phases.
Identify different types of research questions.
Develop a preliminary research question based on identified community problems/concerns.
Session 2: Research methods
Conduct a basic literature review based on working research questions.
Identify different types of research designs.
Identify the best research design for working research questions.
Explain how translation and dissemination fit into the overall research paradigm.
Session 3: Building sustainable academic research partnerships
Identify contributions that community members and academic researchers bring to the partnership.
State the benefits of partnerships to community organizations and academic researchers.
Describe administrative academic requirements that may impact progress.
Identify key working agreements and processes that should be included in partnership development.
Identify specific strategies to promote trust and manage conflict.
Session 4: Program evaluation and research
Describe the goal of program evaluation.
Develop a basic logic model for program evaluation.
Identify scenarios in which program evaluation and research are needed.
Session 5: Funding (Part 1)
List organizational factors that need to be in place before applying for and receiving funding.
List potential funding sources that are relevant to their community organizations.
Initiate a funding announcement query within relevant funding agency databases.
Complete tasks needed to fulfill common preapplication requirements.
Session 6: Funding (Part 2)
List key steps in developing a high-quality proposal.
Demonstrate confidence in writing different proposal components.
Identify key budget development steps.
List strategies to promote efficient postaward management.
Community member training session learning objectives
Session 2: Types and phases of research and methods
Identify different types of research designs.
Gain an understanding of the impact of patient/community stakeholder feedback.
Explain how translation and dissemination fit into the overall research paradigm.
Session 3: Emerging roles and opportunities
Articulate the current and emerging roles that stakeholders can play in the research process.
Explain the different phases of research in relation to community/stakeholder engagement.

Session 1—Combined session for community members and community-based organizations.

## Discussion

We have highlighted how our CE efforts, over a period of several years, led to the development of formal research curricula for community members and CBOs. We employed an iterative, multilayered process to garner feedback and advice from community members and CBO stakeholders. The type of feedback gained during this process, and examples of how it was integrated into the final curricula, may be useful to those wanting to develop and/or tailor research training curricula for their own communities.

Like others who have developed community research training curricula [7, 12, 13], our Community Research Capacity-Building Team relied on an advisory body, the CERC Community Advisory Council, to provide guidance on training content. We took additional steps and solicited and received input on 4 major areas before implementing training: (1) training topic priorities; (2) training format; (3) training logistics; and (4) training compensation. Gathering feedback beyond training topics, the most common type sought from community stakeholders, helps address potential shortcomings that might otherwise only be revealed during post-training evaluations. For example, implementing weekly sessions was a major reason for attrition in 1 community research training program [13], though it is not known whether trainees were queried about frequency preferences before training implementation. In our case, we asked community members and CBO representatives about their preferred training frequencies before training. While no amount of pretraining feedback will guarantee 100% attendance and engagement during implementation, anticipating needs and responding to preferences demonstrates investment which may improve training impact beyond simply removing logistical barriers.

Two of the above four feedback categories were resolved by community member guidance: format and compensation. Relative to training format, community members’ guidance resolved numerous conversations our team had about the pros and cons of combining community members and CBOs for the first training session, “Research 101.” As noted in the Results section, they preferred the combined session and we followed that guidance.

Supported by literature showing that monetary incentives positively impact training attendance [12], our team members initially agreed that compensation be offered but not on the amount. CBO feedback suggested that payment might not be appropriate if attendees were participating on behalf of their organizations. Their guidance helped define the compensation structure, giving CBO trainees and their organization’s autonomy on how to allocate compensation. Being able to solicit this type of guidance resolved team member differences in opinions and increased our confidence in the potential acceptability of our training approaches. When possible, such strategies can be used in finalizing implementation decisions.

While integrating some feedback exactly as proposed by community members and CBOs (e.g., biweekly sessions were preferred by CBOs and proposed for the final curricula), we also incorporated priorities and preferences as part of specific training sessions. For example, “conducting a literature search” was the lowest CBO training priority so we included it as a learning objective under the “Research Methods” training session and “additional resources,” which included local sources for literature search assistance. This strategy may be particularly useful in addressing topics that are not given the highest priority or for which devoting entire training sessions may not be necessary or practical, but are still important to understanding the research process.

Beyond the comprehensive and innovative curricula development process, additional strengths and innovations are noteworthy. Our Community Research Capacity-Building team includes CBO representatives that were vital to workgroup discussions and for synthesizing and incorporating information received from all methods. Both co-authors PL and YV agreed to serve as facilitators during subsequent training curricula piloting. Our ability to solicit feedback at all phases benefitted immensely from our existing CEnR infrastructure and community partnerships. For example, CBO and community member representatives included those that had participated in our previous research capacity-building efforts [9] and as experts in our CE studios [10], respectively.

A constraint on the development process was the amount of time taken to gather and integrate feedback. The iterative nature of the work (e.g., soliciting training priorities, developing and then receiving feedback on training outlines based on those preferences) extended the timeline. Being responsive to early CERC Community Advisory Council feedback meant developing curricula for 2 different stakeholder groups (community members and CBOs), adding to the timeline in curriculum development. A related challenge was time needed to include learning objectives that might address trainees with different research-related experience and expertise. This timeline could certainly be shortened by others if training goals and infrastructure for soliciting feedback already exist, particularly if only 1 curriculum is being developed. Others will still need to weigh the initial time investment in soliciting pretraining feedback against the potential value it holds in promoting training acceptability, effectiveness, and sustainability.

## Conclusion

Engaging diverse community members in an iterative and multilayered mix of quantitative and qualitative methods can tailor research curricula to the needs and preferences of community stakeholders. These efforts proved to be useful in anticipating factors that often influence curricula acceptability and effectiveness.
